# Genomic Characterization and Comparison of Multi-Regional and Pooled Tumor Biopsy Specimens

**DOI:** 10.1371/journal.pone.0152574

**Published:** 2016-03-24

**Authors:** Je-Gun Joung, Joon Seol Bae, Sang Cheol Kim, HyunChul Jung, Woong-Yang Park, Sang-Yong Song

**Affiliations:** 1 Samsung Genome Institute, Samsung Medical Center, Seoul, Republic of Korea; 2 Department of Pathology, Samsung Medical Center, Seoul, Republic of Korea; 3 Departments of Pathology, Sungkyunkwan University School of Medicine, Suwon, Republic of Korea; 4 Molecular Cell Biology, Sungkyunkwan University School of Medicine, Suwon, Republic of Korea; University of Michigan, UNITED STATES

## Abstract

A single tumor biopsy specimen is typically used in cancer genome studies. However, it may represent incompletely the underlying mutational and transcriptional profiles of tumor biology. Multi-regional biopsies have the advantage of increased sensitivity for genomic profiling, but they are not cost-effective. The concept of an alternative method such as the pooling of multiple biopsies is a challenge. In order to determine if the pooling of distinct regions is representative at the genomic and transcriptome level, we performed sequencing of four regional samples and pooled samples for four cancer types including colon, stomach, kidney and liver cancer. Subsequently, a comparative analysis was conducted to explore differences in mutations and gene expression profiles between multiple regional biopsies and pooled biopsy for each tumor. Our analysis revealed a marginal level of regional difference in detected variants, but in those with low allele frequency, considerable discrepancies were observed. In conclusion, sequencing pooled samples has the benefit of detecting many variants with moderate allele frequency that occur in partial regions, but it is not applicable for detecting low-frequency mutations that require deep sequencing.

## Introduction

With the approval of several molecular-targeted therapies, personalized therapeutic approaches have become more practical for clinical cancer care. In general, the implementation of targeted therapies is based on genetic alterations leading to tumor progression in individuals. However, intratumoral heterogeneity hinders precise genetic profiling by lowering the probability of detecting target variations [1]. Tumor tissues taken from the same tumor might harbor different mutations or exhibit distinct phenotypic characteristics [2, 3]. Intratumoral heterogeneity can act as a determinant of treatment failure and disease recurrence [4]. Despite this knowledge, surgically resected tumor specimens are usually divided into several aliquots in the biobank without taking into account regional heterogeneity [5].

Regional genetic heterogeneity of tumor tissues is typically investigated by single-cell genome analysis [2] or targeted deep sequencing [6]. Intratumoral heterogeneity at the single nucleotide level has shown that many mutations are common to several regions, while several other mutations are present only within a single region, suggesting ongoing regional clonal evolution [3, 7]. At the transcriptome level, a recent study indicated that overall mRNA expression profiles in esophageal squamous cell carcinoma (ESCA) specimens are similar in all intratumor comparisons based on microarray-based expression profiling [8]. Minimal regional heterogeneity at the level of the transcriptome might suggest that clonal evolution is not caused by transcriptional control in ESCA. However, single cell transcriptome analysis has revealed expression heterogeneity in glioblastoma, breast cancer, and prostate cancer [1, 9, 10]. RNA sequencing (RNA-seq) on single cells in lung cancer tissue showed high heterogeneity, which was related to cell-specific responses to drug treatments.

Creation of a biobank requires the collection and storage of high-quality biological samples that represent all of a patient’s genetic variation. Recommendations for specimen collection and handling have been developed for clinical trials. A biobank may be defined as the long-term storage of biological samples for research or clinical purposes. Best practices for the management of research biobanks vary according to institutions and international regulations and standards. However, there are many agreed-upon best practices for establishing a biobank for the custodianship of high-quality specimens and data [11]. Although the importance of genetic heterogeneity in patient tumor tissue is increasing, the need for sampling and storage guidelines that reflect the regional variability of mutations remains.

The recent advent of next-generation sequencing (NGS) technologies has led to attempts to identify appropriate therapeutic applications based on high-resolution mutation assessments. Somatic mutational heterogeneity raises the issue of more careful decision-making with the clinical implementation of deep sequencing. Multi-regional analysis through deep sequencing has the potential to overcome the bias related to biopsy from a single region. Basically, pooling of biopsies from a single tumor can significantly reduce sequencing cost and time, but the applicability in clinical sequencing has not been studied in diverse cancer types. More reliable assessment is needed in tumor sequencing strategies. We examined genomic and transcriptomic profile differences between multiple regions and pooling of samples. A comparative analysis of genomic and transcriptomic profiles using whole-exome sequencing (WES) and RNA-seq data, respectively, revealed that multiple regional sampling is the most suitable technique for addressing genetic variability in cancer.

## Materials and Methods

### Sample preparation and design for multi-regional differences in genetic profiles

**Fig 1:**
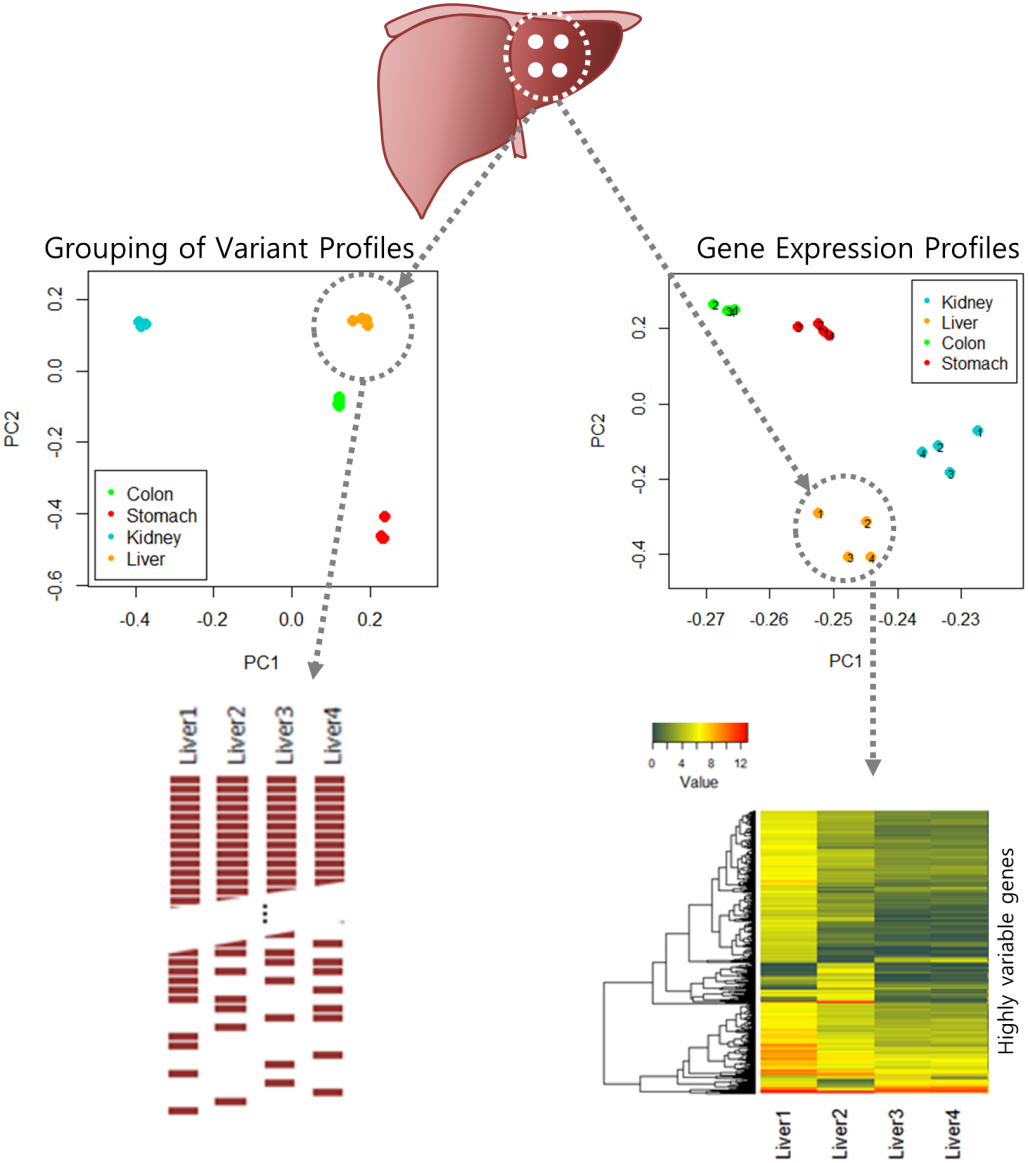
Schematic overview of the comparison of mutation and expression patterns from multiregion sequencing of whole-exome and whole-transcriptome. Regional samples of each cancer were closely grouped over 2-D space based on principal component analysis (PCA). The differences in genomic variants and in gene expression between sequencing are shown.

Surgical specimens were stored in 3–4 aliquots depending on tumor size. We typically analyzed one aliquot for genomic and biochemical characterization of the tumor. To measure the genetic variability of different aliquots in the biobank, we designed an experiment comparing the genomic and transcriptome profiles of pooled samples against multiple regional samples (Fig 1). We selected one case each from four types of cancer including hepatocellular carcinoma, stomach adenocarcinoma, renal cell carcinoma, and colon adenocarcinoma from the Biobank of Samsung Medical Center (SMC). Each surgical sample for genome analysis was obtained from four different tumor foci falling with the same distance. Four tumor foci were chosen according to the following criteria: 1) Each region corresponded to a vertex of a square; 2) The length of each edge was 2 cm; and 3) Each fraction had the same volume. Once pooled sample was prepared from four tumor foci, nucleic acids were extracted by QIAamp DNA mini kit (Qiagen, Valencia, CA, USA). The frozen tumor samples were macro-dissected and lightly stained with hematoxylin and eosin (H&E) to identify regions consisting of ≥ 30% cancer cells. We compared genomic profiles of WES and RNA-seq from four aliquots of each cancer type (Fig 1). Mixed samples were also generated *in silico* by choosing random reads from individual samples. This study was exempted from IRB approval because it was conducted as a part of a quality check of specimens stored in the Samsung Medical Center Biobank.

### Genomic DNA and total RNA extraction

Tissue frozen in nitrogen solution was crushed into a small volume using a sterilized surgical knife, and then homogenized using the Tissue Lyzer II (Qiagen, Valencia, CA, USA) at 20 Hz for 30 seconds. After the homogenized tissue was digested by proteinase K at 56°C for 16 hours, genomic DNA and total RNA were extracted using the AllPrep DNA/RNA mini kit (Qiagen) according the manufacturer’s protocol. The quality and quantity of genomic DNA were determined using NanoDrop 8000 UV-Vis spectrometer (Thermo Scientific, DE, USA), Qubit 2.0 Fluorometer (Life technologies, Grand Island, NY, USA) and 2200 TapeStation Instrument (Agilent Technologies, Santa Clara, CA, USA). Total RNA quality and quantity were also determined using a Nanodrop 8000 UV-Vis spectrometer (Thermo Scientific) and Lab-on-a-Chip on an Agilent 2100 Bioanalyzer (Agilent Technologies).

### Whole exome sequencing

Genomic DNA (1 μg) from each tissue sample was sheared using a Covaris S220 (Covaris, MA, USA) and used for the construction of a library using the SureSelect XT Human All Exon v5 and the SureSelect XT reagent kit, HSQ (Agilent Technologies) according to the manufacturer’s protocol. This kit was designed to enrich 335,756 exons of 21,058 genes, covering ~71 Mb of the human genome. After enriched exome libraries were multiplexed, the libraries were sequenced on the HiSeq 2500 sequencing platform (Illumina). Briefly, a paired-end DNA sequencing library was prepared through gDNA shearing, end-repair, A-tailing, paired-end adaptor ligation, and amplification. After hybridization of the library with bait sequences for 16 hours, the captured library was purified and amplified with an index barcode tag, and the library quality and quantity were measured. Sequencing of the exome library was carried out using the 100-bp paired-end mode of the TruSeq Rapid PE Cluster kit and TruSeq Rapid SBS kit (Illumina).

### Whole transcriptome sequencing

The library construction for whole transcriptome sequencing was performed using Truseq RNA sample preparation v2 kit (Illumina). Isolated total RNA (2 μg) was used in a reverse transcription reaction with poly (dT) primers using the SuperScript^™^ II reverse transcriptase (Invitrogen/Life Technologies) according to the manufacturer’s protocols. Briefly, an RNA sequencing library was prepared through cDNA amplification, end-repair, 3’ends adenylate, adapter ligation, and amplification. Quality and quantity of library were measured by Bioanalyzer and Qubit. Sequencing of the transcriptome library was carried out using the 100-bp paired-end mode of the TruSeq Rapid PE Cluster kit and TruSeq Rapid SBS kit (Illumina).

### Exome-sequencing data analysis

The sequencing reads were aligned to the UCSC hg19 reference genome (downloaded from http://genome.ucsc.edu) using Burrows-Wheeler Aligner (BWA)[12], version 0.6.2 with default settings. PCR duplications were marked using Picard-tools-1.8 (http://picard.sourceforge.net/), data cleanup was followed by Genome Analysis Toolkit (GATK)[13] and variants were identified with MuTect (http://www.broadinstitute.org/cancer/cga/mutect) and LoFreq (http://sourceforge.net/projects/lofreq/) under default parameters. Perl script and Annovar were used to annotate variants. The detected SNVs were filtered with cancer-associated 337 genes using cancer panels. Variants with > 90% variant allele frequency (VAF), defined as homozygous germline SNPs, were filtered out. Also, we filtered out germline variants satisfying the following three criteria: 1) SNPs reported in dbSNP; 2) Variants with >1% frequency in population using ESP5400 and 1000 Genome data; 3) Variants not reported in COSMIC and TCGA data. We evaluated mapping quality using the Picard tool (broadinstitute.github.io/picard). PCA analysis was performed for a profile of detected variants using the princomp function of the R tool. In order to test with *in silico* data of four multi-regional samples, we generated the mixed sample by randomly choosing 25% reads from each sample. The correlation between the average variant allele fraction of the regional samples and the variant allele fraction of the pooled sample (or the *in silico* mixed sample) was measured using Pearson’s correlation coefficient. Copy number variations were identified by comparing mapped read counts between tumor and control mixing other 12 normal blood samples, and then we performed the segmentation procedure with DNAcopy R package.

### RNA-sequencing data analysis

The reads from the FASTQ files were mapped against the hg19 human reference genome using TopHat version 2.0.6 (http://tophat.cbcb.umd.edu/). The output files in BAM format were analyzed using HTSeq version 0.6.1 [14] to quantify the transcript abundance. A total of 18,161 coding genes were selected to measure the transcript abundance and then low expressed genes were filtered out based on a maximum read count > 20 across all samples. Read counts obtained from mapping to genes were normalized via the TMM (Trimmed Mean of M-values) normalization method. Pairwise scatter plots of log2 expression values between samples were generated using the “pairs” R function and Pearson’s correlation coefficients of gene expression profiles were measured between samples in order to determine the difference in gene expression profiles among them. In order to measure the variability between samples at the gene level, the fold change in gene expression between both regions (or each region and pooled sample) was calculated. For any comparison between the samples, genes that passed the fold change cut-off were selected for fraction calculation. The sequencing data analyzed in this manuscript have been deposited in the NCBI's Sequence Read Archive (SRA) and are accessible through accession number SRP066596 (http://www.ncbi.nlm.nih.gov/sra/SRP066596).

## Results

### Comparison of single nucleotide variations

**Fig 2:**
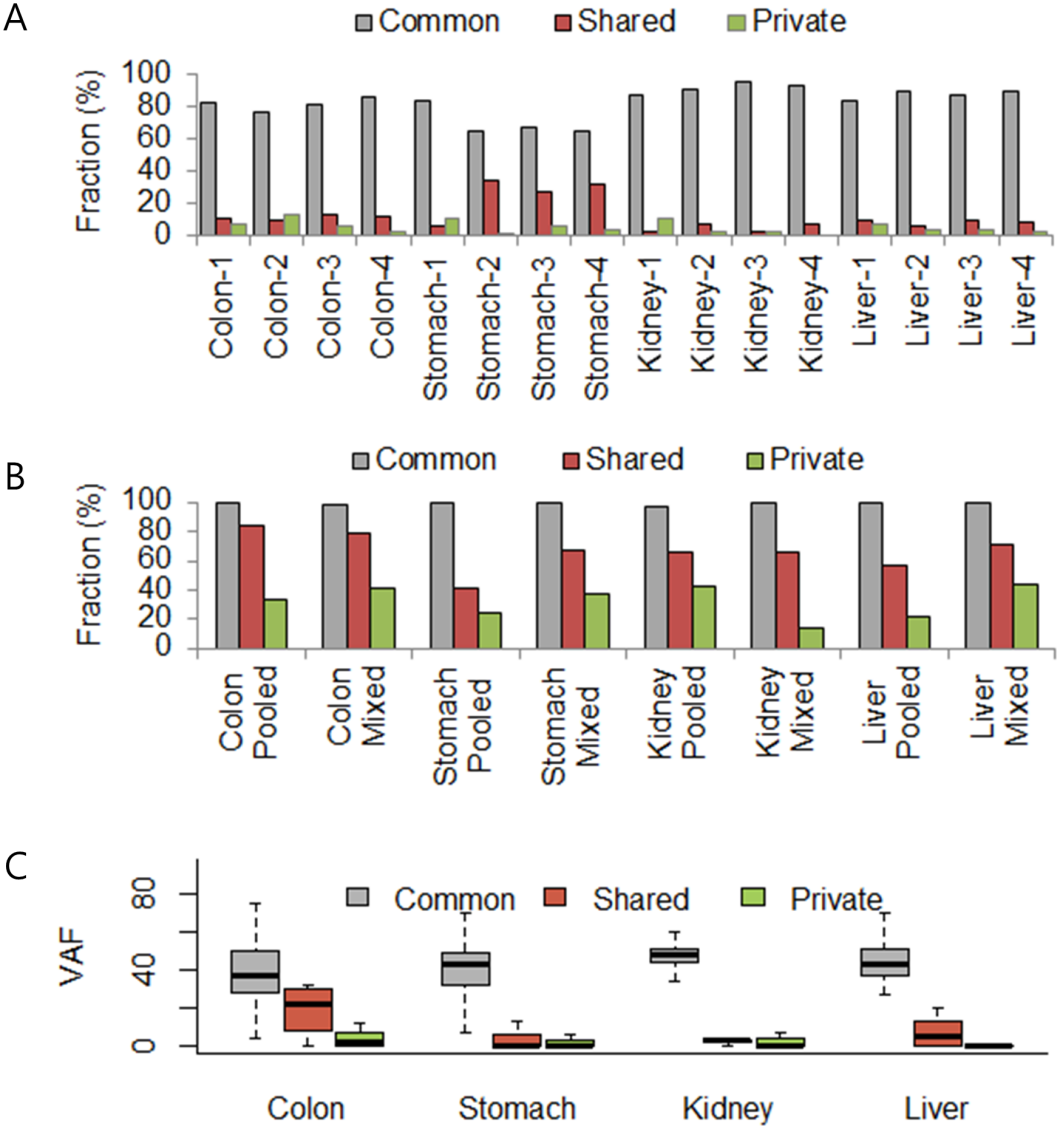
The characteristics of common, shared and private variants from regional samples. (A) Fractions of common, shared, and private variants identified among different regions. (B) Fractions of common, shared, and private variants identified from pooled- or mixed-samples. (C) Variant allele frequency (VAF) distribution of common, shared, and private variants identified among different regions.

Whole exome sequencing on all samples produced 125.9±13.7 million reads with target coverages of 136.7±17.4x (S1 Table). Single nucleotide variations (SNVs) from four “regional” samples of each tumor type were called by MuTect and LoFreq (S3 Table). SNVs in each regional sample were classified into three groups: i) common variants found in all four regional samples; ii) shared variants detected in > 2 samples; and iii) private variants detected in a single sample. Although most of the variants were common in four regions, the proportion of common variants was quite variable in four cases. More than 53.9% of detected SNVs were concordant among all four regional stomach cancer samples, while renal cell carcinoma showed the highest concordance rate (79.6%) in four regional samples (Fig 2A). Likewise, stomach cancer and colon cancer case showed a marked number of private variants (16/102 and 36/152), which might indicate the genetic heterogeneity in these samples.

We tested whether sample pooling from multiple regions could improve variant detection. The number of variants found in pooled samples increased in all four cancer types, as shared as well as private variants were detected (Fig 2A and 2B). In particular, most of the common variants in the multiregional samples could be detected in pooled samples (>97.4%; Fig 2B). In addition, shared and private variants were detected by sample pooling (Fig 2B). SNVs from the pooled and mixed samples were concordant with shared SNVs in the multi-regional samples from 40 to 85%. Private SNVs of a single region, which are expected to have an approximate detection rate of 25% (1 of 4 samples), were detected with a higher than average detection rate of 30.9%. However, basically pooled biopsy missed many private SNVs or some common SNVs. It missed 5.6%, 0.6%, 3.7% and 2.8% of SNVs with moderate VAF (with > 20% VAF at least in one regional sample among shared variants) detected from regional biopsies for colon, kidney, stomach and liver, respectively (S1 Fig). The missed SNVs were more presented at low VAFs.

**Fig 3:**
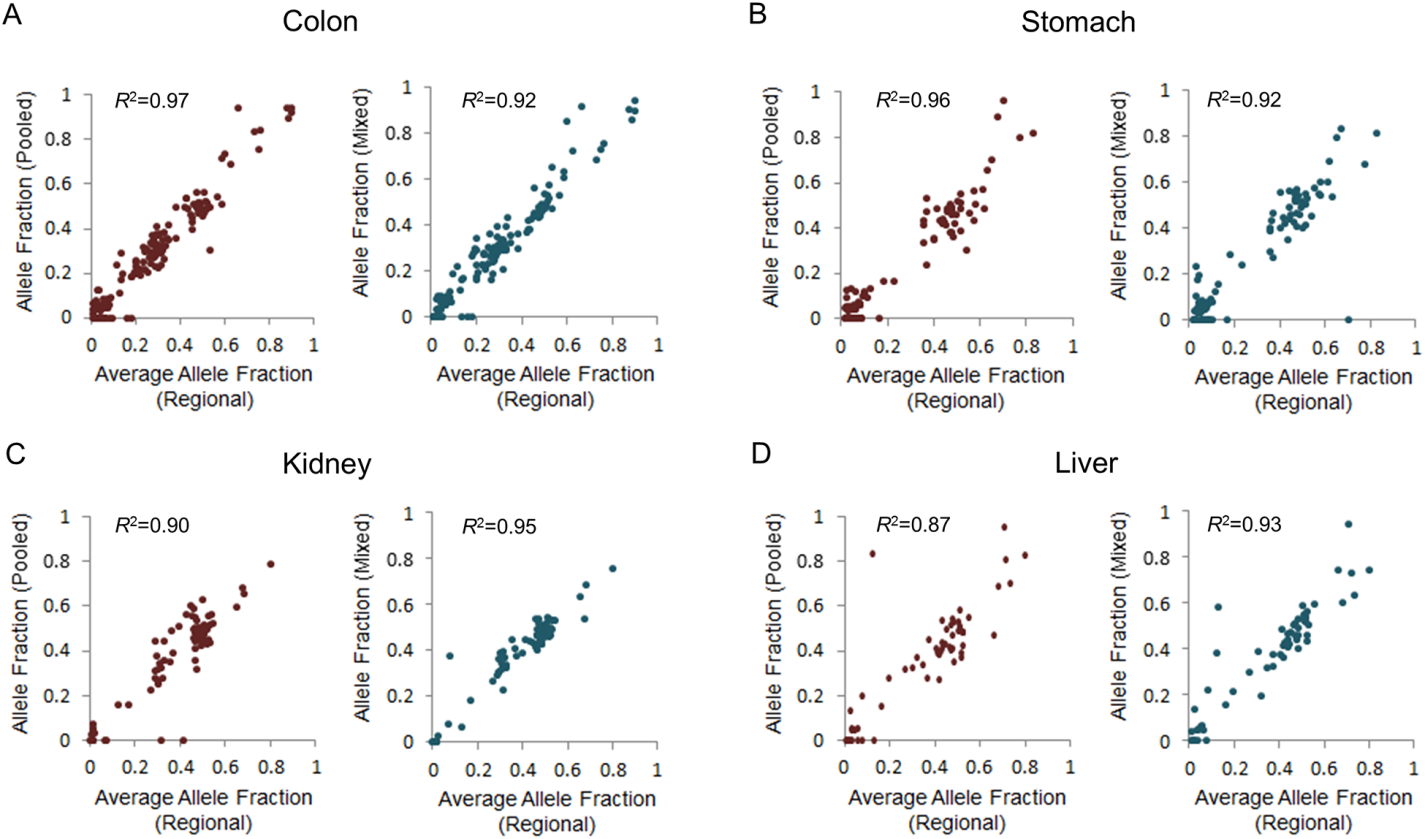
Correlation between the average variant allele fraction of regional samples and the allele fraction of pooled sample (or the *in silico* mixed sample).

**Fig 4:**
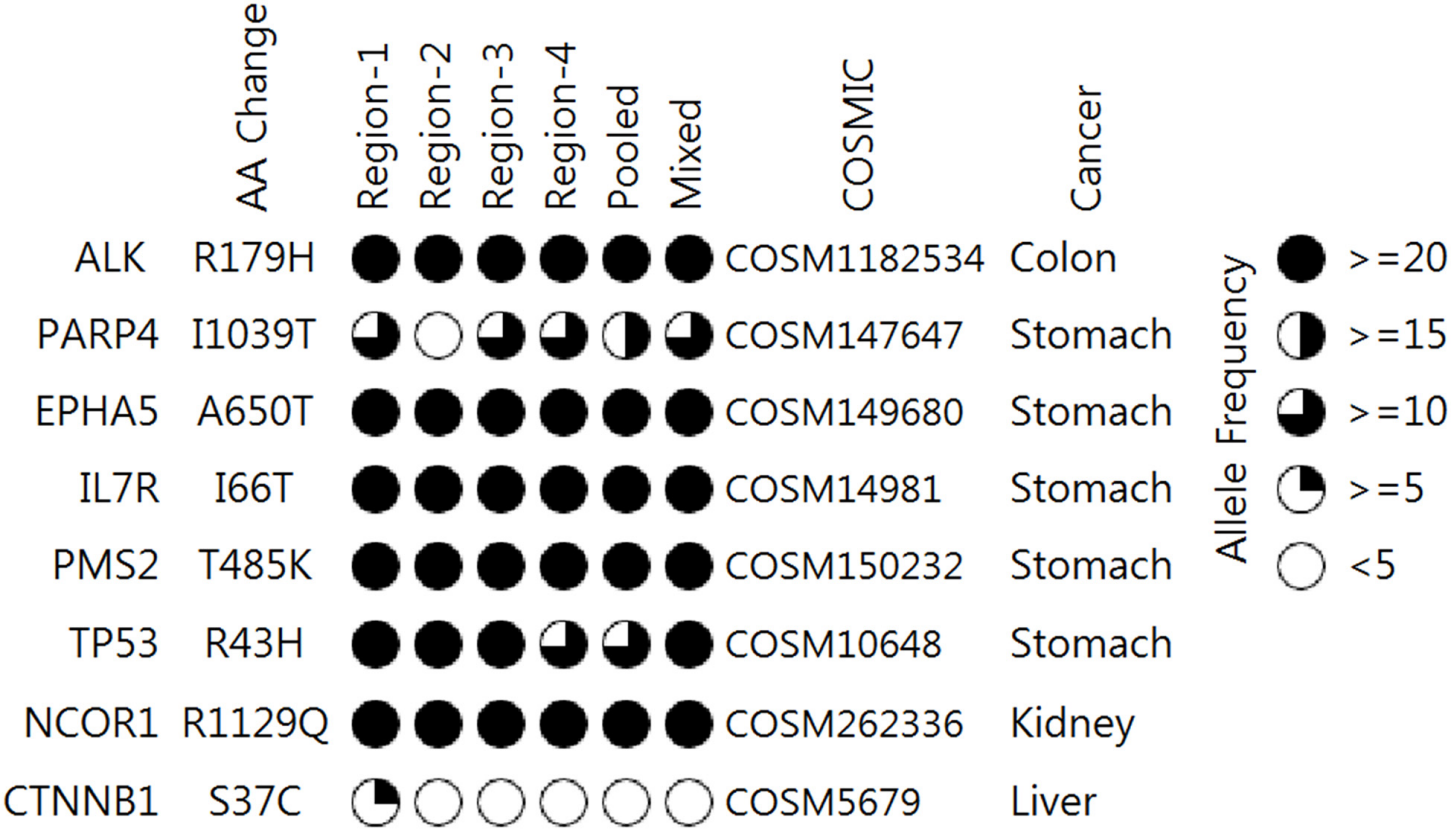
Profile of variants listed on the COSMIC DB and labeled as the same tissue type. The fraction of each circle indicates tumor variant allele frequency.

We measured the distribution of variant allele frequency (VAF) for common, shared, and private variants from regional samples (Fig 2C). VAF of common variants detected from four regions was higher than that of shared and private (median was near 40). However, shared or private variants were detected at low allele frequencies. The variant allele frequency of each pooled sample was similar overall to the average allele frequency of four regional samples (Fig 3). Depending on VAFs, the correlations of the pooled (mixed) samples were 0.97 (0.92), 0.96 (0.92), 0.90 (0.95) and 0.87 (0.93) for colon, kidney, stomach and liver samples, respectively. When we selected variants listed in the COSMIC database and labeled them as the same cancer type (Fig 4), most were common variants, while a missense variant in *PARP4* of stomach cancer was a shared variant and *CTNNB1* of liver cancer was a private variant. These results conclusively demonstrated the limitation of single or pooled samples to detect all of the variants present in patient specimens.

### Comparison of gene expression profiles

**Fig 5:**
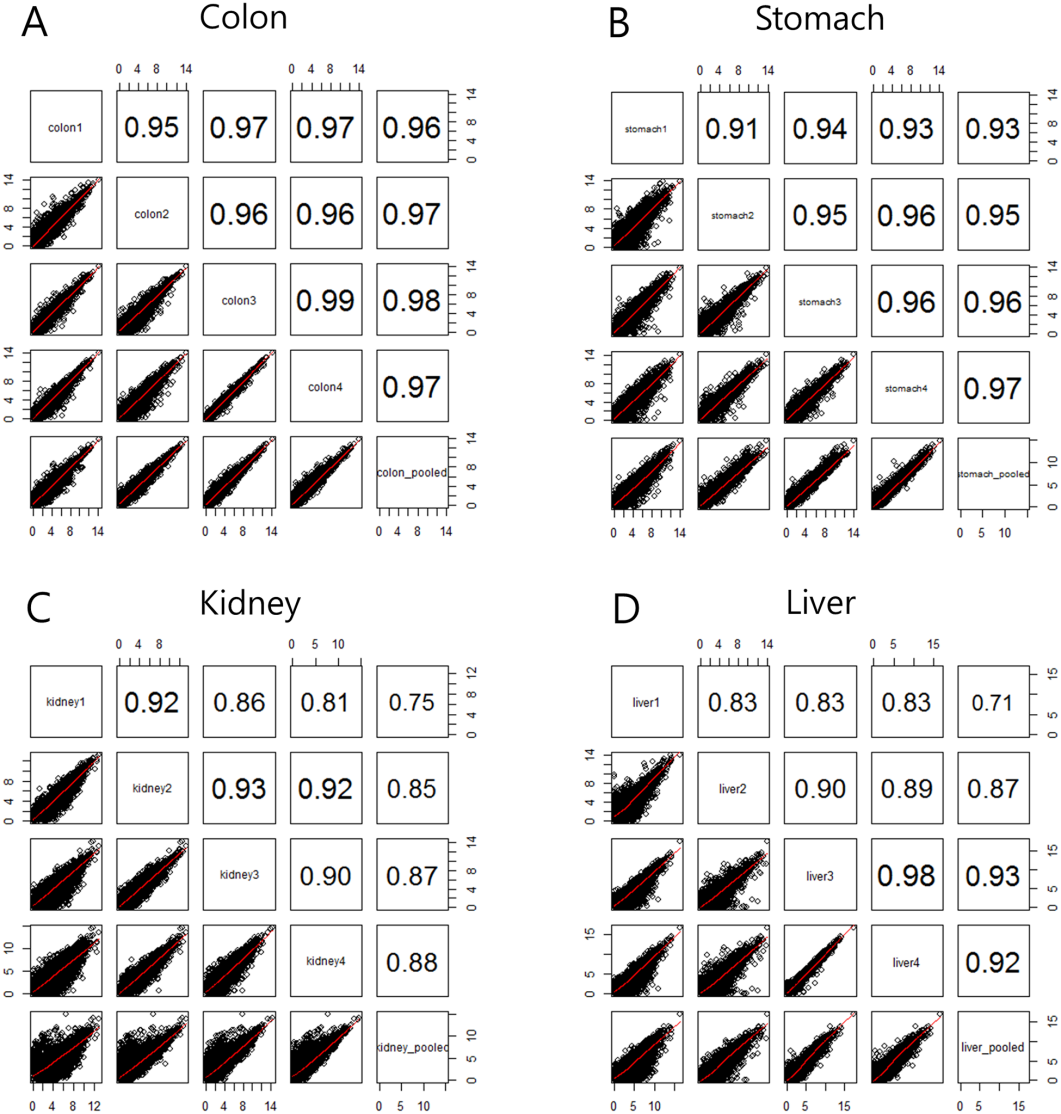
Pairwise correlations and scatter plots of expression profiles from whole transcriptome sequencing data. Pearson’s correlation coefficients included all coding genes.

Across all RNA-seq data from 20 samples, 89±2.1% of total reads were uniquely aligned to the human genome reference (S2 Table). We obtained expression profiles of 17,145 coding genes from the mapped reads (S4 Table). Tumor purity estimation was performed based on transcriptional profile from RNA-seq using ESTIMATE [15]. Tumor purities were more than 60% for all the samples and the variation between regional samples was not high (*S<* = ±10) (S2 Fig). In order to determine the magnitude of difference in gene expression between regional samples, we calculated pair-wise correlation between samples (Fig 5). The average correlation between the different regional samples was *R*^2^ = 0.97 (colon), *R*^2^ = 0.94 (stomach), *R*^2^ = 0.89 (kidney), and *R*^2^ = 0.88 (liver), respectively. The gene expression profiles of colon and stomach samples have a higher correlation than those of kidney and liver samples. Intratumoral heterogeneity at the level of RNA transcripts could be higher in kidney and liver than in colon and stomach. The average pairwise correlation between each regional sample and pooled sample was *R*^2^ = 0.97 (colon), *R*^2^ = 0.95 (stomach), *R*^2^ = 0.84 (kidney), and *R*^2^ = 0.86 (liver), respectively, indicating that the gene expression of pooled samples was similar overall to that of each regional sample. The regional samples from kidney and liver cancers showed relatively more variable gene expression profiles.

**Fig 6:**
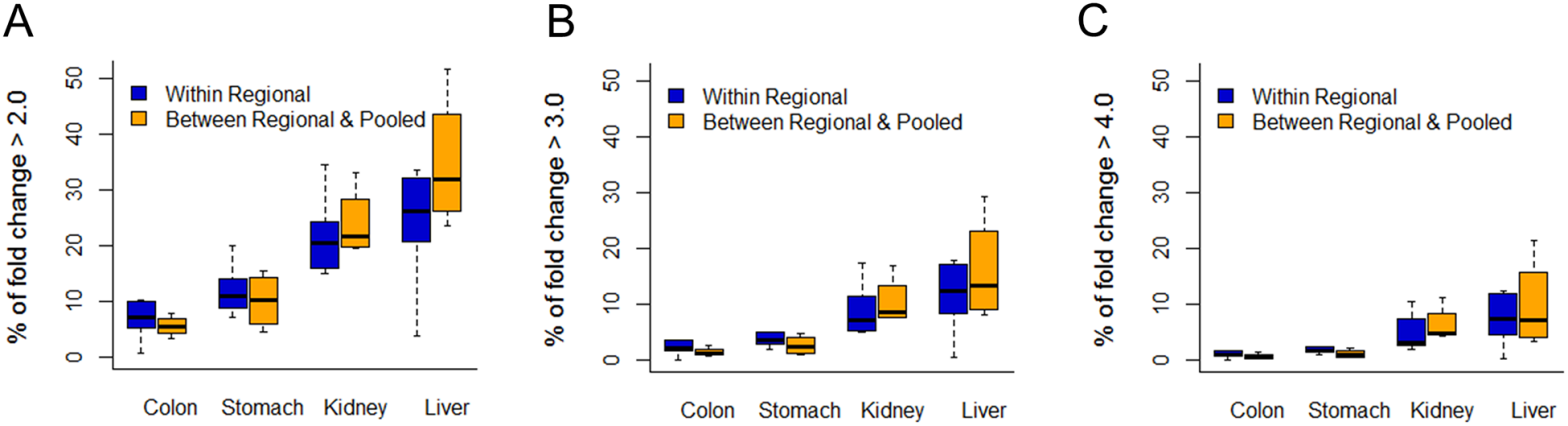
Percent of differentially expressed genes in multiregional samples (blue) and between the pooled sample and regional samples (orange). Each bar represents the distribution of the fraction of genes showing fold change difference in expression ((A) > 2-fold, (B) > 3-fold, and (C) > 4-fold) between each pair of samples. For every possible comparison between the four regional samples (a total of six comparisons) and between the four regional samples and the pooled sample (four comparisons) in each tumor case, genes showing a difference in fold change were selected for fraction calculation.

In order to determine how much variability exists between samples at the gene level, the gene expression profiles within regional samples were further compared based on the fold change difference (> 2–4 fold) between each pair of samples (Fig 6). The similarity in gene expression profiles within multiple regions was quite different in cancer cases. Colon cancer showed similar gene expression profiles, while regional samples of liver cancer were significantly different from each other. For example, the *HRAS* oncogene showed a 5-fold expression difference between the four regions of liver cancer. The gene expression profiles of the pooled samples were also similar to those of multi-regional samples in colon and stomach samples. These results demonstrate that overall expression patterns are similar between different regions, and between regional and pooled samples, but some genes show a highly variable expression pattern.

## Discussion

Genome analysis of cancer requires high-quality human cancer tissue in order to obtain the most accurate results [16]. Protocols for sample acquisition for biobanks should include a procedure to assessthe genetic heterogeneity by sequencing without compromising the pathological diagnosis [17]. In particular, any actionable variant in a refractory cancer patient can be used to personalize treatment with target drugs based on the genomic profile. We have compared the analytical performance of WES and RNA-seq in pooled and multiple regional samples. Sequencing of multiregional samples could cover a higher number of variants. Because the cost of next-generation sequencing is decreasing, we need to consider multiple sampling from a single tumor specimen to ensure minor variations are represented. Biopsies of multiple sites will be more beneficial in metastatic cancers, as the subclones within the primary tumor can be also presented in metastatic sites. Therefore the clinical treatment should focus on these subclones with metastatic potential [5].

Understanding the actual distribution of variants and gene expression profiles presented in bulk tumor specimens has been a challenge due to tumor heterogeneity. Our analysis indicated that most detected variants were highly concordant across regions; however, some discrepancies were observed for variants with low allele frequency. For example, we found an apparent discrepancy in the presentation of recurrent somatic mutations of *CTNNB1* (D32H and S37C) in liver cancer (Fig 4). They were private variants detected in only one region and S37C of both mutations was not detected from the pooled sample due to low allele frequency (expected VAF < 6% for D32H and < 2% for S37C). Mutations in *CTNNB1* are considered to be cancer drivers for HCC development [18]. In an experimental model of HCC, *CTNNB1*, *IGF1R*, *FGF19*, *CCND1* and *IGF2* have been evaluated in the oncogenic addiction loop, but this study has yet to enter the advanced clinical developmental phase [19]. This example suggests that a single biopsy is not sufficient to determine individualized cancer therapy, specifically considering clinically relevant genomic alterations. In addition, the sequencing of pooled samples should be addressed to ensure detection of low allele frequency mutations.

Our transcriptome analysis indicated that mRNA expression was relatively unstable when comparing the expression profiles between multiple regions in cancers. This regional difference in expression may be caused by functional heterogeneity of subclones during tumor progression. In addition, copy number pattern is overall similar for all regional samples, but many regional differences exist at focal regions in chromosome, indicating the presence of regional tumor heterogeneity (S3 Fig). Copy number alterations showing remarkable regional differences were observed in the following regions: absence of 16q amplification in Colon-4, chromosome 12 amplification in Kidney-4, and 13q amplification in Stomach-1. Those regions may affect the gene expression, and gene expression changes can be underestimated in single biopsy. A single biopsy may fail to estimate representative gene expression in certain tumors. Our analysis suggested that pooled samples of multiple regions also did not reduce the bias in measuring precise gene expression.

Further studies are required to resolve technical issues and perform other in depth analyses. For example, although most variance between different regional biopsies from the same tumor was due to intratumoral heterogeneity, some of the variation was due to technical problems arising during the biopsy process. Thus, the interpretation of sequencing results should be carefully and systematically conducted. Another example is the expression consistency per gene category across samples. As some genes are consistently expressed across a tissue while other genes are highly variable, different expression profiles over regions of the tumor may be exhibited in a specific gene class.

Our study has still some limitations, including sample size and subclone estimation issues. The number of test samples is important for achieving accurate assessment. Although only one set per tumor type was examined in this study, our preliminary results may provide clues to a sequencing strategy that would enable efficient genomic profiling of samples in tumor tissue banks. This pilot study will be extended in a larger sample size to better understand and characterize the regional tumor heterogeneity. In addition, the clonal status of each mutation will be estimated from mutation and copy number profiles with tumors and their matched normal samples.

We demonstrated that different mutation profiles and gene expression patterns within a single tumor were observed in four cancer types. Several clinical applications including different drug sensitivity, functional impact on tumor progression and resistance to therapy can be developed based on the genomic and transcriptomic patterns of multiple regions. In conclusion, our analysis suggests that a single biopsy might not be sufficient to determine personalized cancer therapy, and sequencing from pooled samples should be improved for precise identification of genomic variants from distinct regions.

## Supporting Information

S1 Fig The detection limitation in pooled biopsy for SNVs detected in regional biopsies. (TIF)

S2 Fig The tumor purity estimation of samples. (TIF)

S3 Fig The copy number profile of regional and pooled samples. (TIF)

S1 Table Summary of whole-exome sequencing data. (XLSX)

S2 Table Summary of whole-transcriptome sequencing data. (XLSX)

S3 Table The list of identified SNV. (XLSX)

S4 Table Gene expression values from whole-transcriptome sequencing. (XLSX)

## References

[1] Patel AP, Tirosh I, Trombetta JJ, Shalek AK, Gillespie SM, Wakimoto H, et al Single-cell RNA-seq highlights intratumoral heterogeneity in primary glioblastoma. Science. 2014;344(6190):1396–401. 10.1126/science.1254257 24925914 PMC4123637

[2] Navin N, Kendall J, Troge J, Andrews P, Rodgers L, McIndoo J, et al Tumour evolution inferred by single-cell sequencing. Nature. 2011;472(7341):90–4. 21399628 10.1038/nature09807 PMC4504184

[3] Gerlinger M, Rowan AJ, Horswell S, Larkin J, Endesfelder D, Gronroos E, et al Intratumor heterogeneity and branched evolution revealed by multiregion sequencing. N Engl J Med. 2012;366(10):883–92. 10.1056/NEJMoa1113205 . 22397650 PMC4878653

[4] Bedard PL, Hansen AR, Ratain MJ, Siu LL. Tumour heterogeneity in the clinic. Nature. 2013;501(7467):355–64. . 24048068 10.1038/nature12627 PMC5224525

[5] Swanton C. Intratumor heterogeneity: evolution through space and time. Cancer Res. 2012;72(19):4875–82. 10.1158/0008-5472.CAN-12-2217 23002210 PMC3712191

[6] Kumar A, Boyle EA, Tokita M, Mikheev AM, Sanger MC, Girard E, et al Deep sequencing of multiple regions of glial tumors reveals spatial heterogeneity for mutations in clinically relevant genes. Genome Biol. 2014;15(12):530 10.1186/s13059-014-0530-z 25608559 PMC4272528

[7] Gerlinger M, Horswell S, Larkin J, Rowan AJ, Salm MP, Varela I, et al Genomic architecture and evolution of clear cell renal cell carcinomas defined by multiregion sequencing. Nat Genet. 2014;46(3):225–33. . 24487277 10.1038/ng.2891 PMC4636053

[8] Yan W, Shih J, Rodriguez-Canales J, Tangrea MA, Player A, Diao L, et al Three-dimensional mRNA measurements reveal minimal regional heterogeneity in esophageal squamous cell carcinoma. The American journal of pathology. 2013;182(2):529–39. 10.1016/j.ajpath.2012.10.028 23219752 PMC3562732

[9] Lee MC, Lopez-Diaz FJ, Khan SY, Tariq MA, Dayn Y, Vaske CJ, et al Single-cell analyses of transcriptional heterogeneity during drug tolerance transition in cancer cells by RNA sequencing. Proceedings of the National Academy of Sciences of the United States of America. 2014;111(44):E4726–35. 10.1073/pnas.1404656111 25339441 PMC4226127

[10] Wyatt AW, Mo F, Wang K, McConeghy B, Brahmbhatt S, Jong L, et al Heterogeneity in the inter-tumor transcriptome of high risk prostate cancer. Genome Biol. 2014;15(8):426 10.1186/s13059-014-0426-y 25155515 PMC4169643

[11] Hallmans G, Vaught JB. Best practices for establishing a biobank. Methods Mol Biol. 2011;675:241–60. 10.1007/978-1-59745-423-0_13 . 20949394

[12] Li H, Durbin R. Fast and accurate short read alignment with Burrows-Wheeler transform. Bioinformatics. 2009;25(14):1754–60. Epub 2009/05/20. 10.1093/bioinformatics/btp324 [pii]. 19451168 PMC2705234

[13] McKenna A, Hanna M, Banks E, Sivachenko A, Cibulskis K, Kernytsky A, et al The Genome Analysis Toolkit: a MapReduce framework for analyzing next-generation DNA sequencing data. Genome Res. 2010;20(9):1297–303. Epub 2010/07/21. 10.1101/gr.107524.110 gr.107524.110 [pii]. 20644199 PMC2928508

[14] Anders S, Pyl PT, Huber W. HTSeq—a Python framework to work with high-throughput sequencing data. Bioinformatics. 2015;31(2):166–9. 10.1093/bioinformatics/btu638 25260700 PMC4287950

[15] Yoshihara K, Shahmoradgoli M, Martinez E, Vegesna R, Kim H, Torres-Garcia W, et al Inferring tumour purity and stromal and immune cell admixture from expression data. Nat Commun. 2013;4:2612 Epub 2013/10/12. ncomms3612 [pii]. 24113773 10.1038/ncomms3612 PMC3826632

[16] Esgueva R, Park K, Kim R, Kitabayashi N, Barbieri CE, Dorsey PJ Jr., et al Next-generation prostate cancer biobanking: toward a processing protocol amenable for the International Cancer Genome Consortium. Diagn Mol Pathol. 2012;21(2):61–8. 22555088 10.1097/PDM.0b013e31823b6da6 PMC4123125

[17] Carmignani L, Picozzi S, Casellato S, Bozzini G, Marenghi C, Macchi A, et al A proposed new technique in prostate cancer tissue bio-banking: our experience with a new protocol. Pathol Oncol Res. 2012;18(3):663–8. 10.1007/s12253-011-9492-6 . 22215310

[18] Tornesello ML, Buonaguro L, Tatangelo F, Botti G, Izzo F, Buonaguro FM. Mutations in TP53, CTNNB1 and PIK3CA genes in hepatocellular carcinoma associated with hepatitis B and hepatitis C virus infections. Genomics. 2013;102(2):74–83. 10.1016/j.ygeno.2013.04.001 . 23583669

[19] Villanueva A, Llovet JM. Impact of intra-individual molecular heterogeneity in personalized treatment of hepatocellular carcinoma. Hepatology. 2012;56(6):2416–9. 10.1002/hep.26124 . 23212778

