# Supplementary figures and images for: Genomic Characterization and Comparison of Multi-Regional and Pooled Tumor Biopsy Specimens
Source: PLoS One. 2016 Mar 24;11(3):e0152574. doi: 10.1371/journal.pone.0152574 (PMC4807092; doi:10.1371/journal.pone.0152574)

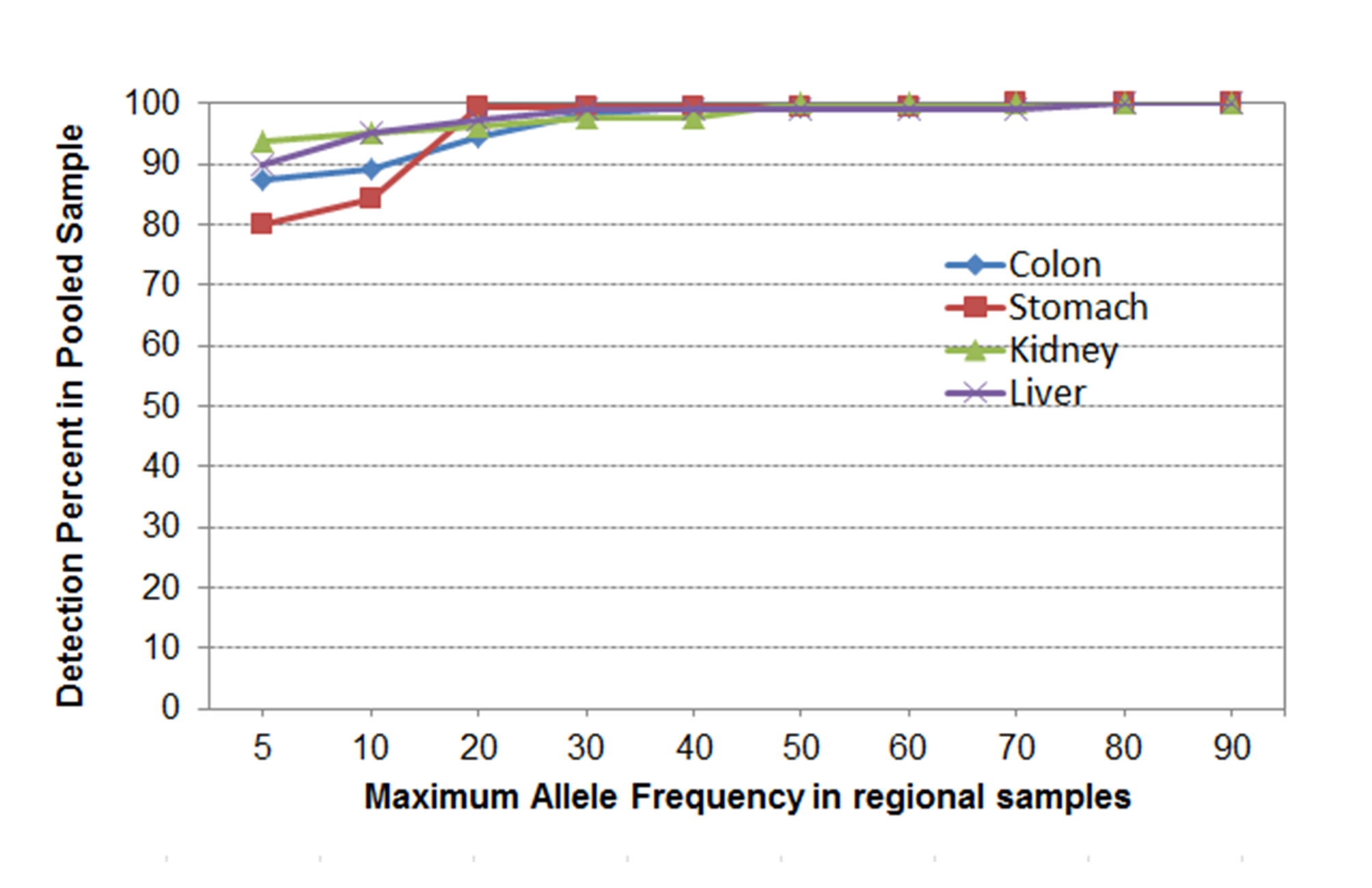

Supplement: S1 Fig — (TIF) [file pone.0152574.s001.tif]

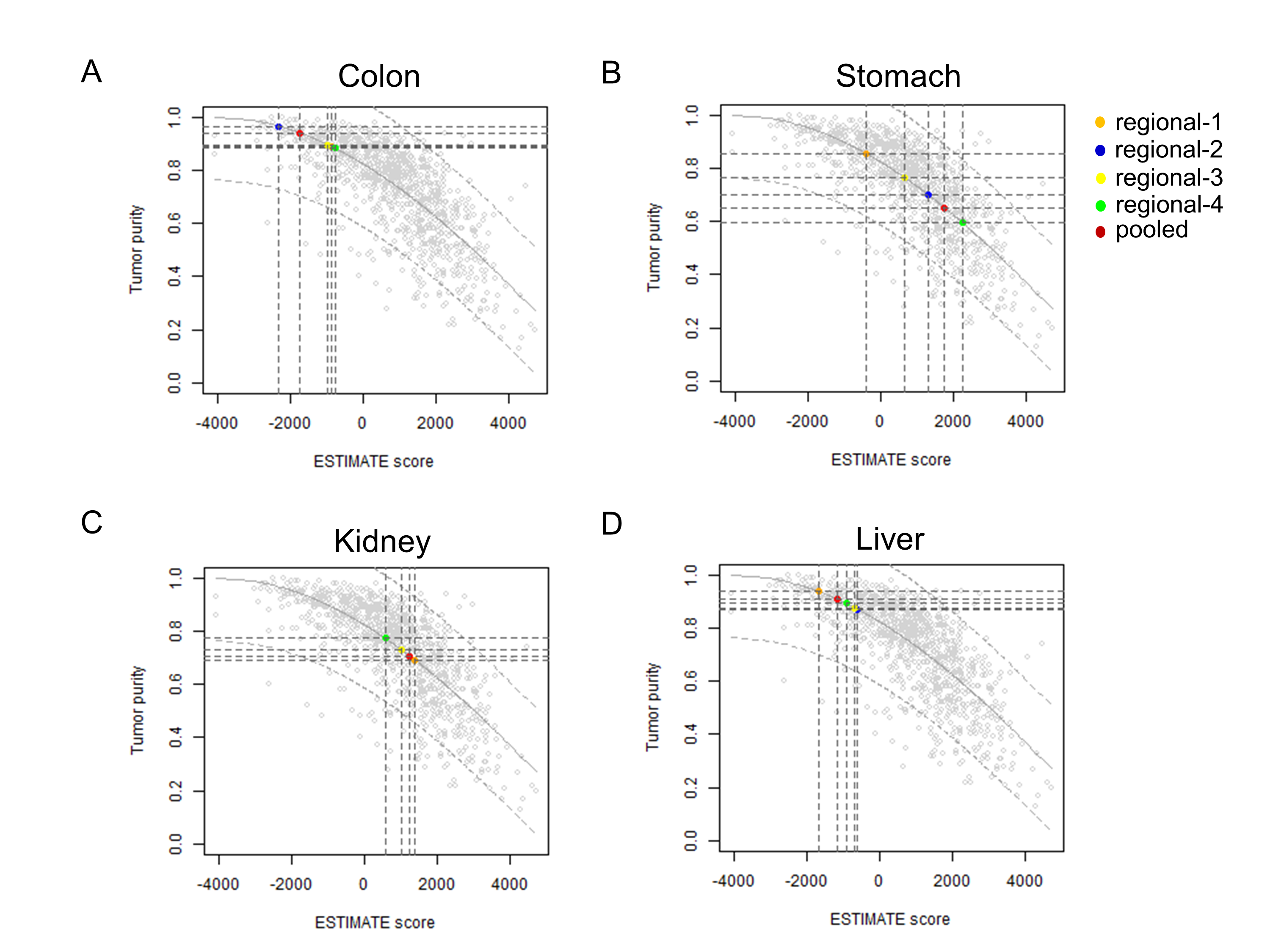

Supplement: S2 Fig — (TIF) [file pone.0152574.s002.tif]

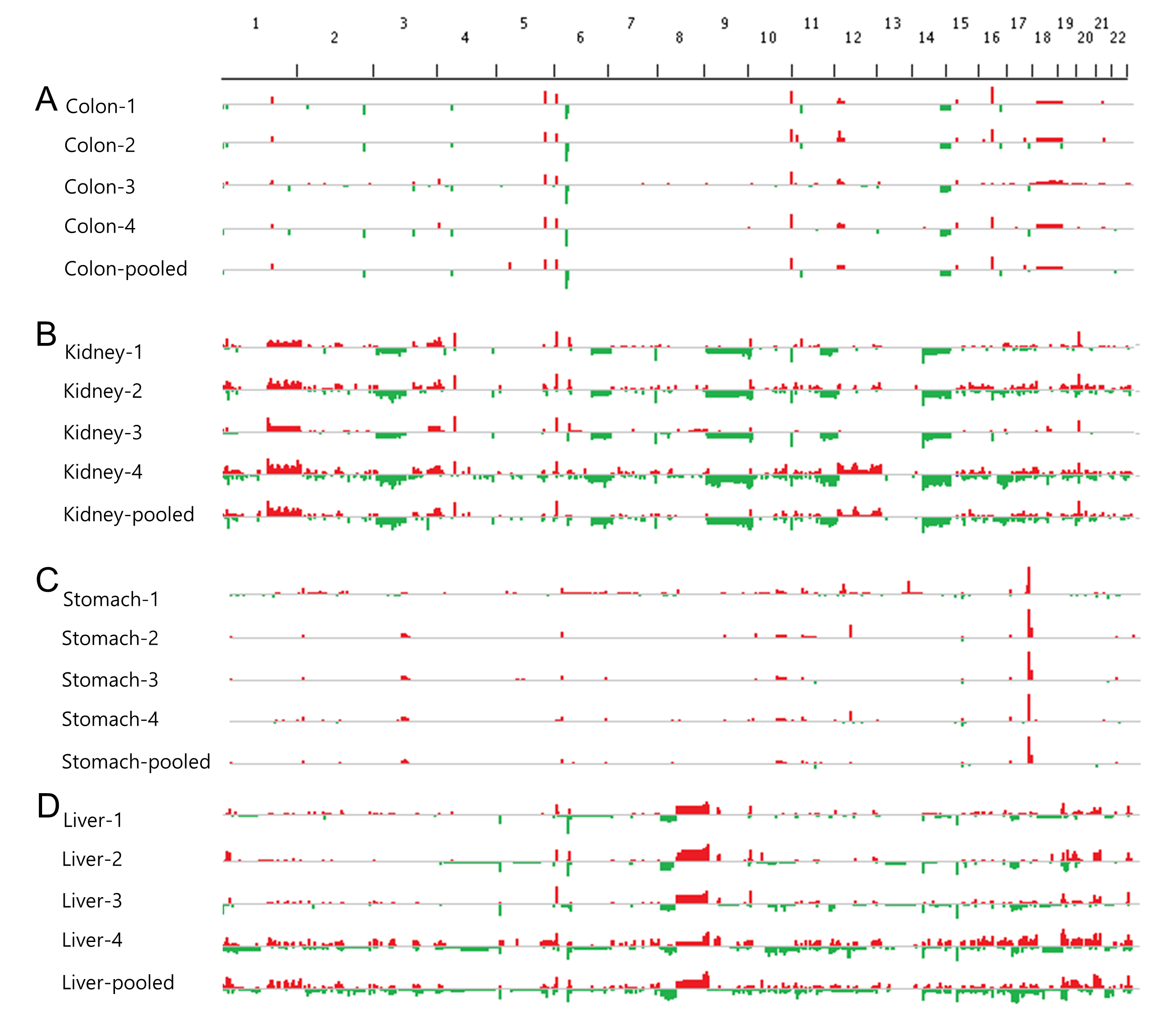

Supplement: S3 Fig — (TIF) [file pone.0152574.s003.tif]
